# Clinical Outcomes of May–Thurner Syndrome in Pediatric Patients: A Single Institutional Experience

**DOI:** 10.1055/s-0040-1714694

**Published:** 2020-08-20

**Authors:** Deepti M. Warad, Amulya Nageswara Rao, Haraldur Bjarnason, Vilmarie Rodriguez

**Affiliations:** 1 Division of Pediatric Hematology-Oncology, Department of Pediatric and Adolescent Medicine, Mayo Clinic, Rochester, Minnesota, United States; 2 Special Coagulation Laboratory, Division of Hematopathology, Mayo Clinic, Rochester, Minnesota, United States; 3 Division of Vascular and Interventional Radiology, Department of Radiology, Mayo Clinic, Rochester, Minnesota, United States

**Keywords:** May–Thurner syndrome, deep vein thrombosis, pediatric, children

## Abstract

**Introduction** May–Thurner syndrome (MTS) is a vascular anatomic variant resulting in compression of the left common iliac vein by the right common iliac artery, affecting approximately 22% of the population. In adults, following acute deep vein thrombosis (DVT) of the iliofemoral veins, the incidence of postthrombotic syndrome (PTS) and recurrent DVT are high if treated with anticoagulation alone, warranting adjunctive treatment with thrombolysis and stent placement. However, there is paucity of literature documenting the course of treatment and associated outcomes in pediatric patients with MTS.

**Methods** A retrospective chart review of pediatric patients (≤ 18 years of age) with radiologic confirmation of MTS with or without DVT evaluated and/or treated at our institution from January 1, 2005 through December 31, 2015 was conducted.

**Results** Seventeen patients (4 male; 13 female) were identified. Median age was 15.4 years (range 8.8–17.1 years) with a median follow-up of 1.2 years (range 0.4–7.5 years). Thirteen (76.5%) patients presented with left lower extremity DVT. Management included catheter-directed thrombolysis ( *n* = 5), systemic thrombolysis ( *n* = 1), and mechanical thrombectomy ( *n* = 1). Fifteen patients were treated with anticoagulation including two patients with endovascular stents without DVT. Median duration of anticoagulation was 6.3 months (range 3.2–18.7 months). Ten patients (59%) underwent stent placements.

Complete and partial thrombus resolution was noted in six patients each and no resolution in one patient. Four patients had recurrence/progression of thrombus ( *n* = 3 with stents) at a median time of 29 days (range 12–495 days). No bleeding complications were observed. Clinically documented or self-reported PTS was noted in 8 patients (62%).

**Conclusion** There are no clear guidelines for MTS management in children and adolescents. In our cohort, thrombolysis, anticoagulation, or stent placements were not associated with bleeding risks, with recurrence/progression of DVT and signs and symptoms of PTS noted in 30 and 62%, respectively. Further studies are needed to determine a standardized treatment approach of the pediatric patient with MTS with or without thrombosis.

## Introduction

May–Thurner syndrome (MTS) also known as Cockett syndrome is an anatomic variant resulting in compression of the left iliac vein by the overlying right common iliac artery against the lumbar vertebrae. As the vessel is compressed, changes of chronic endothelial irritation including collagen deposition can develop over time with partial or complete occlusion of the vein. This anatomic variant associated with MTS is known to increase the incidence of left-sided lower extremity deep vein thrombosis (DVT). 1 2 3 4 The true prevalence of MTS is unknown, but it is estimated to be associated in 18 to 49% of left leg DVT. 1 2 Clinically, most patients present with acute DVT in the left lower extremity or progressively unilateral leg swelling and pain without an identifiable thrombosis; however, many patients may remain asymptomatic throughout their lifetime. 3 The clinical association of DVT and MTS is relatively low with a reported range of 2 to 3%. 5

The main treatment goal of MTS associated with iliofemoral DVT is directed toward prevention of postthrombotic syndrome (PTS) (e.g., swelling secondary to venous insufficiency, pain, skin discoloration, and venous stasis skin ulceration). Anticoagulant therapy with unfractionated heparin (UFH), low molecular weight heparin (LMWH), with or without transition to warfarin, or direct-acting anticoagulants (e.g., rivaroxaban, apixaban) is the main treatment modality for patients presenting with acute DVT. Anticoagulation prevents thrombus propagation and/or pulmonary embolism (PE) but does not resolve the chronic venous occlusion secondary to thrombus or anatomic occlusion of the iliac vein secondary to compression by the overlying right iliac artery. Acute thrombosis associated with MTS has been historically treated with catheter-directed thrombolysis (CDT), mechanical thrombectomy (CDT in combination with mechanical thrombectomy is often referred to as pharmacomechanical thrombectomy [PMT]), percutaneous balloon angioplasty, and stenting. 5 6 7 8 In adults with MTS and DVT, recurrence of thrombosis is more common in those patients treated with anticoagulation alone 9 than those treated with PMT. Most adults with DVT in the setting of MTS undergo stent placement 10 11 whether in combination with PMT or later in association with venous recanalization of the chronic postthrombotic obstruction, angioplasty, and stent placement. However, few case reports and studies have reported the course of treatment and outcomes in pediatric patients with MTS. 12 13 14 There are currently no established standard treatment approaches in pediatric patients with MTS.

The purpose of our study was to retrospectively review our institutional management of pediatric MTS with or without DVT, and outcomes including thrombus response, recurrence/progression of thrombus, stent failure, and development of PTS.

## Methods

This study was approved by the Mayo Clinic Institutional Review Board. Medical record data was abstracted for all pediatric patients (≤ 18 years of age) who presented for evaluation and/or treatment of MTS with or without DVT. The study period comprised from January 1, 2005 to December 31, 2015. Patient demographic data, baseline thrombosis risk factors (e.g., body mass index [BMI]), congenital or acquired thrombophilia, immobility, and oral contraceptive pill (OCP) use, were reviewed. Overweight was defined as a BMI ≥ 25 kg/m ^2^ and obesity as a BMI ≥ 30 kg/m ^2^ . 15 Thrombophilia testing included antiphospholipid antibodies (i.e., lupus anticoagulant immunoglobulin [Ig] G, IgM, and anticardiolipin antibodies), activated protein C (APC) resistance with reflex gene sequencing for factor V Leiden if APC resistance abnormal, prothrombin G20210A mutation, antithrombin activity and antigen, protein C activity, free protein S, fibrinogen level, and fibrin D-dimer. Radiological techniques used for diagnosis of MTS and DVT, modality of treatment (e.g., catheter or systemic thrombolysis, thrombectomy, balloon angioplasty, stent placement), duration, and type of anticoagulation were also collected. Thrombosis outcomes were defined as complete resolution (no residual thrombus identified by ultrasound Doppler, magnetic resonance venography [MRV], or computed tomography venography [CTV], venography), partial resolution (residual thrombosis), and no resolution (complete occlusion of one or more affected left lower extremity veins). Recurrence and progression of DVT were defined as per guidelines developed by the Perinatal and Pediatric Haemostasis Subcommittee of the Scientific and Standardization Committee of International Society of Thrombosis and Haemostasis. 15

Stent failure was defined as no demonstrable blood flow secondary to thrombosis or mechanical failure if no thrombosis. Signs and symptoms of PTS were reviewed from medical-clinical notes (e.g., pain, swelling, dilated blood vessels, varicosities, and skin ulceration). A previously developed and validated PTS survey instrument was mailed to patients with thrombosis. 16 17

### Statistical Analyses

Standard statistical methods were used to summarize collected data: frequency and percent for categorical variables and mean, median, and range for ordinal or continuous variables. Analysis was performed using JMP software (JMP version 13, SAS Institute Inc, Cary, North Carolina, United States; 1989–2016). Comparative analysis among subgroups and significance of difference (two-tailed *p* -value < 0.05) were reported if sample size allowed analysis. Contingency analysis by Fisher's exact test was used for categorical comparisons and survival analysis was performed by Kaplan–Meier test.

## Results

### Demographics

Seventeen patients (13 female) were identified. Median follow-up was 1.2 years (range 0.4–7.5 years) ( Table 1 ). Fifteen patients identified as Caucasian, one patient each identified as African American and Hispanic. Median age at diagnosis of MTS was 15.4 years (range 8.8–17.1 years).

**Table 1:** Patient characteristics, management, and outcomes

Case ID	Gender/Age in years	Risk factors	Location extent thrombosis	Thrombolysis	Stenting	Anticoagulation	Recurrence/Progression (time)	Thrombosis outcomes (CR/PR/NR)	PTS/duration follow-up (y)
1	F/16.7	OCP	Inferior IVC/external and internal left iliac veins/common femoral extending popliteal and tibial veins	Catheter-directed Alteplase (0.5 mg/h, 1 mg/h)	Yes	UFH/LMWH/Warfarin/Clopidogrel	No	PR	No (5.6)
2	F/16.2	OCP	Left common iliac/common femoral, upper deep femoral to popliteal/PE	Catheter-directed Alteplase (0.5 mg/h)	Yes	UFH/Warfarin LMWH recurrent occlusion stent	Yes (6 d)	PR	No (1.2)
3	F/15.5	Heterozygous factor V Leiden/immobility and OCP	Left iliofemoral to popliteal	None	Yes	UFH/Warfarin/Clopidogrel	No	PR	No (0.4)
4	F/15.2	OCP/ overweight (BMI 25.6)	Mid to distal left common iliac vein and external iliac vein	None	No	Warfarin/Clopidogrel	No	CR	Yes (0.9)
5	F/14.6	OCP	NA	None	No	None	No	NA	NA
6	M/14.5	None	Left common iliac vein through common femoral to popliteal	Catheter-directed Alteplase (0.5 mg/h, 0.75 mg/h)	No	LMWH/Warfarin	Yes (11 d)	PR	No (3.0)
7	M/17.1	Heterozygous factor V Leiden Overweight (BMI 29.34)	Common femoral, superficial femoral to popliteal and proximal calf veins/PE	Systemic t-PA (0.03 mg/kg/h, 0.06 mg/kg/h)	No	UFH/LMWH/Rivaroxaban	No	PR	Yes (1.2)
8	F/11.6	None	Left iliac vein	None	Yes	LMWH	No	CR	Yes (7.5)
9	F/16.4	Heterozygous factor V Leiden/Immobility/OCP	Left common iliac, femoral, deep femoral, and popliteal	Catheter-directed thrombolysis outside institution	Yes	LMWH/Warfarin	Yes (104 d)	NR	Yes (0.4)
10	M/8.8	None	NA	None	No	None	No	NA	NA
11	F/13.6	None	NA	None	Yes	Rivaroxaban	No	NA	NA
12	M/14.8	None	Left common and external iliac and femoral	None	No	ASA initially then LMWH/Warfarin after balloon venoplasty	No	PR	Yes (0.7)
13	F/14.6	Heterozygous factor V Leiden, protein C deficiency, and OCP	Left common and external iliac and common femoral	Mechanical thrombectomy-outside institution	Yes	LMWH/Warfarin	Yes (20 d)	CR	Yes (3.2)
14	F/15.4	Immobility and OCP Obese (BMI 30.66)	Left external iliac/common femoral to popliteal/PE	Catheter-directed Alteplase (0.5 mg/h)	Yes	LMWH/Warfarin	No	CR	Yes (1.1)
15	F/16.5	None	NA	None	Yes	Heparin/LMWH/ Warfarin/Rivaroxaban and clopidogrel after Nutcracker repair	No	NA	NA
16	F/16.2	Immobility and OCP Overweight (BMI 29.4)	Left external iliac and common femoral	None	Yes	LMWH/Warfarin/Rivaroxaban	No	CR	Yes (1.0)
17	F/16.8	Homozygous factor V Leiden/OCP Obese (BMI 33.01)	Left common femoral to posterior tibial/PE	None	No	LMWH/Warfarin	No	CR	No (0.8)

Abbreviations: ASA, acetylsalicylic acid; BMI, body mass index; CR, complete resolution; DVT, deep vein thrombosis; LMWH, low molecular weight heparin; NA, not applicable; NR, no resolution; OCP, oral contraceptive pills; PE, pulmonary embolism; PR, partial resolution; PTS, postthrombotic syndrome; UFH, unfractionated heparin.

### Clinical Presentation

**Fig. 1:**
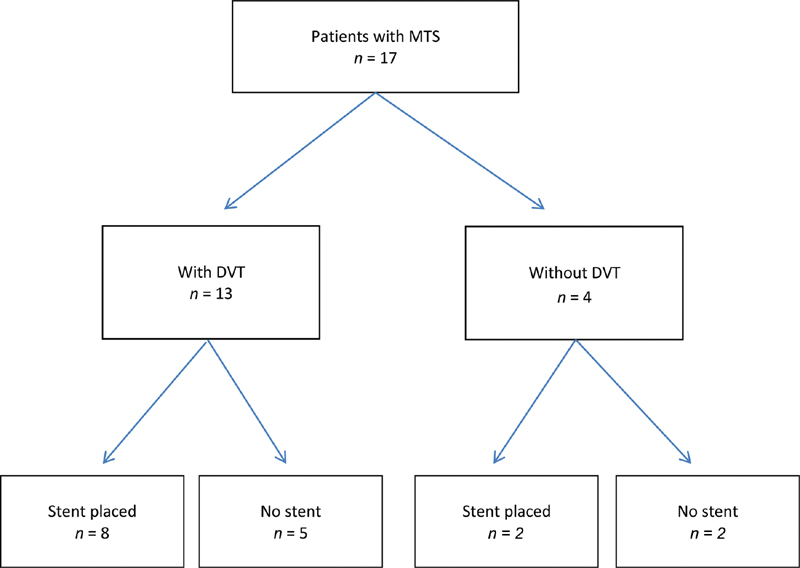
Summary of patients with May–Thurner syndrome.

Pain and swelling were the most common presenting symptoms ( *n* = 13, 76%). Thirteen patients presented with acute DVT of the left lower extremity (76%) while four were diagnosed with MTS without DVT (asymptomatic sibling of MTS patient [ *n* = 1] and pain and/or swelling [ *n* = 3]) ( Fig. 1 ). Four patients were noted to have PE at the time of DVT diagnosis ( Table 1 ).

### Thrombotic Risk Factors

Comorbidities associated with risk for thrombosis such as increased BMI (≥ 25 kg/m ^2^ ), immobility, OCP use, and thrombophilia were analyzed among patients with or without DVT ( Table 1 ). The median BMI was 23.63 kg/m ^2^ (range 18.32–33.01 kg/m ^2^ ). The median BMI was similar at 23.81 kg/m ^2^ when four patients without DVT were excluded (range 18.44–33.01 kg/m ^2^ ). Thrombophilia testing was performed in only six patients. Five patients tested positive for factor V Leiden mutation (heterozygous = 4; homozygous = 1). Ten out of the 13 (76%) patients with DVT had one or more thrombosis risk factors such as inherited thrombophilia, immobility, increased BMI, and/or use of OCP. Increased BMI ( *n* = 5), immobility ( *n* = 4), and OCP use ( *n* = 10) as risk factors for thrombosis were found to be nonsignificant ( *p* -value 0.26, 0.52, and 0.25, respectively).

### Diagnostics

Radiological modalities used to establish the diagnosis of MTS with or without DVT included: ultrasound Doppler and venography ( *n* = 7), ultrasound Doppler with MRV ( *n* = 5), ultrasound Doppler and CTV ( *n* = 4), and MRV only ( *n* = 1).

### Management

Management in patients with or without DVT is summarized in Table 1 .

#### Initial treatment: Anticoagulation and Thrombolysis

Out of the 13 patients that presented with acute DVT, 6 (35%) were treated with thrombolysis in addition to systemic anticoagulation (catheter-directed = 5; systemic = 1) at a median of 1 day from DVT diagnosis (range 0–8 days). The median treatment duration was 2 days (range 1–4 days). One patient underwent PMT upon diagnosis of DVT at an outside institution 3 years prior to presentation. Overall, choice of anticoagulation therapy was variable and consisted of initial UFH or LMWH that were later transitioned to or continued on LMWH, warfarin, or direct oral anticoagulants such as rivaroxaban. Median duration of anticoagulation was 6.3 months (range 3.2–18.7 months). Antiplatelet agents such as clopidogrel and aspirin were used in some patients ( Table 1 ). Anticoagulation with or without antiplatelet agents were used in patients without DVT who underwent placement of a stent or balloon angioplasty.

#### Endovascular Stenting

Ten patients (59%) had stent placement at a median of 3.5 days (range 0–376 days) in those following DVT diagnosis ( *n* = 8) ( Table 2 ). In two patients without DVT, the stents were placed at 767 and 1,117 days (2.1 and 3.1 years) from the diagnosis of MTS, respectively. Stent failure occurred in 5 patients (50%) at a median of 1.7 months (range 0.3–72 months): 1 due to growth-related stent migration from compression site, 1 due to stent narrowing, and 3 due to thrombosis leading to stent replacement. All 5 patients underwent interventional stent recanalization procedure, of which 4 remained patent while 1 remained occluded. Proportion of stent survival at last follow-up was 90% (9 of 10 stents) with a median follow-up of 1.1 years (range 0.2–6.5 years). There was no association between stent size (diameter of 14 mm vs. > 14 mm) and stent failure ( *p* = 0.52)

**Table 2:** Summary of endovascular stenting and patency rates

Case ID	Thrombosis	Time to stent placement from MTS diagnosis in days (in years)	Stent location	Type of stent (number)	Stent size	Time to stent failure in days (in years)	Additional stent (location)	Stent patency at last follow-up	Duration from MTS diagnosis in months (in years)
1	Yes	4	CIV	Protégé ^TM1^	14 mm × 80 mm	−	NA	Patent	67 (5.6)
			EIV extending into CFV	Protégé ^TM2^	14 mm × 80 mm, 14 mm × 40 mm				
2	Yes	0	CIV	Wallstent ^TM1^	14 mm a	9	Protégé™ (CIV)	Patent	14.3 (1.2)
			EIV extending into CFV	Wallstent ^TM2^	14 mm a , 12 mm a				
3	Yes	0	CIV	Wallstent ^TM1^	18 mm × 60 mm	−	NA	Patent	4.5
8	Yes	337	CIV	Protégé ^TM1^	14 mm × 60 mm	2190 (6 y)	Wallstent™ (IVC)	Patent	89.6 (7.5)
9	Yes	1	CIV	Wallstent ^TM2^	14 mm × 60 mm, 14 mm × 40 mm	112	Not placed	Occluded (CIV, EIV)	5.3
			EIV extending into CFV	Protégé ^TM2^	14 mm × 80 mm, 14 mm × 80 mm				
11	No	1117 (3.1 y)	CIV	Wallstent ^TM1^	16 mm × 60 mm	−	NA	Patent	36.7 (3.1)
			EIV	Wallstent ^TM1^	16 mm × 60 mm				
13	Yes	0	CIV, EIV, and CFV	Unknown	Unknown	31	Type unknown	Patent	38.1 (3.2)
14	Yes	1	CIV	Wallstent ^TM1^	16 mm × 60 mm	−	NA	Patent	13.4 (1.1)
15	No	767 (2.1 years)	CIV	Wallstent ^TM1^	16 mm × 40 mm	51	Not placed	Patent	26.9 (2.2)
16	Yes	287	CIV	Wallstent ^TM2^	14 mm × 60 mm, 14 mm × 40 mm	−	NA	Patent	12
			EIV	Protégé ^TM2^	14 mm × 80 mm, 14 mm × 80 mm				

Abbreviations: CIV, left common iliac vein; CFV, left common femoral vein; EIV, left external iliac vein; IVC, inferior vena cava; MTS, May–Thurner syndrome; NA, not applicable.

Note: Protégé™ [Medtronic, Minneapolis, Minnesota, United States]; Wallstent™ [Boston Scientific, Marlborough, Massachusetts, United States].

a Stent length information not available.

### Outcomes

Complete resolution of thrombus was noted in 6 (46%) patients, partial resolution in 6 (46%), and no response in 1 (8%). Four patients (31%) had recurrence/progression of thrombus ( *n* = 3 with stents) at a median time of 29 days (range 12–495 days). No bleeding complications were observed. One patient had progression of DVT 11 days from diagnosis; CDT was initiated with partial resolution of the thrombosis; no stent was placed due to multiple collateral vessels. The role of thrombolysis therapy and its potential role for the prevention of recurrent thrombosis was analyzed and found to be nonsignificant ( *p* = 1.0). Neither degree of thrombosis clearance (complete vs. partial or no resolution) nor stent implantation (stent vs. no stent) were found to be associated with thrombosis recurrence ( *p* = 0.26 and *p* = 1.0, respectively).

On reviewing patients charts for signs and symptoms of PTS such as swelling, pain, skin discoloration, varicosities, skin ulceration, and claudication, five patients were noted to have had signs and symptoms consistent with PTS at the time of last follow-up. Only 4 patients responded to the mailed PTS survey that confirmed PTS documentation in 1 patient and an additional 3 patients reported PTS symptoms (mild PTS = 1; moderate PTS = 3) resulting in a total of 8 (62%) patients with PTS following DVT at a median follow-up of 16 months (range 9.6–89.8 months). Of the 8 patients with DVT that had stent placements, 5 (63%) patients had clinically documented and/or self-reported PTS at a median follow-up of 17 months (range 4.9–89.8 months). The degree of DVT clearance (complete vs. partial or no resolution), use of thrombolysis, and stent placement were not found to be significantly associated with development of PTS ( *p* = 1.0, 0.10, and 0.62, respectively).

## Discussion

Compression of the left iliac vein by the overriding right iliac artery, also known as MTS, can result in left iliofemoral thrombosis in approximately 50 to 60% of patients as a result of intraluminal spurs from compression. 4 18 Similar to other pediatric series, a female preponderance was noted (M:F ratio 1:3.3). Other precipitating factors such as immobility, inherited thrombophilia, surgery, hormonal therapy, and pregnancy were also noted. 4 19 20 21 In our review, 77% of patients with MTS and DVT had one or more thrombosis risk factors such as inherited thrombophilia, immobility, and/or use of OCPs.

Several radiological imaging modalities are useful in establishing the diagnosis of MTS. Ultrasound Doppler is usually the first and fastest method used to diagnose DVT. 22 However, this radiological technique cannot identify the anatomic compression of the left iliac vein reliably and cannot demonstrate the venous spurs caused by chronic compression. To accurately diagnose MTS, other cross-sectional imaging modalities such as CTV or angiography, magnetic resonance angiography, and/or venography are used for detailed visualization of the pelvic vasculature and anatomy. 23 24 25

**Fig. 2:**
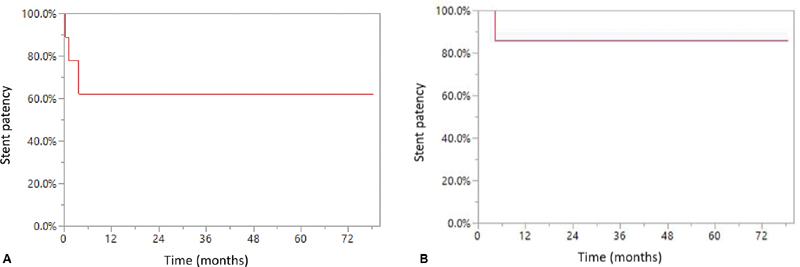
Stent patency. ( **A** ) Primary patency. ( **B** ) Secondary patency.

Anticoagulation is the first-line treatment used to prevent clot extension and occurrence of PE. However, anticoagulation alone neither corrects the anatomic compression of the left iliac vein nor results in clot dissolution. Surgical thrombectomy was considered a treatment option before PMT and stent placements became more commonly used. 26 27 Despite the different surgical techniques, similar primary and secondary patency have been reported at 5 years (42 and 59%, respectively). 28 Due to advances in endovascular techniques, MTS patients rarely have to undergo open vascular surgical procedures. 4 8 10 19 29 In adults, catheter-directed PMT with or without stent placement in addition to anticoagulation are considered the standard treatment for MTS with acute thrombosis with patency rates reported at 79 to 100% at 2 years. 11 29 30 31 32 To date, pediatric (≤ 18 years of age) literature consist primarily of case reports and limited case series, of which the largest series include Goldenberg et al, 12 Murphy et al, 33 and Goldman et al 14 that studied 6 (prospective), 5 (prospective), and 10 (retrospective) cases, respectively. In these reports, all patients presenting with thrombotic MTS received anticoagulation with catheter-directed mechanical or pharmacomechanical thrombolysis followed by endovascular stent placement. Murphy et al and Goldman et al 14 reported primary patency rates of 100% at the end of procedure and 79% at 12 months, respectively, while Goldman et al 14 reported secondary patency rate of 100% at 12 months and 89% at 36 months, as defined by the Society of Interventional Radiology. 34 Primary and secondary patency rates in the 6 patients in Goldenberg study were 40 and 20% at 12 and 24 months, respectively. 12 14 In our series, only 59% patients (10/17) had stent placements. Five stent failures were noted due to thrombosis or mechanical failure. Patency was restored in 3 patients with additional stent placements ( *n* = 3) and CDT ( *n* = 1). A primary patency rate of 62% at 12 months ( Fig. 2A ), with secondary patency rate of 88% at last follow-up ( Fig. 2B ) were observed. The limited sample size precluded definitive comparisons of outcomes based on thrombolytic therapy.

The role of congenital or acquired thrombophilia as a risk factor for thrombosis recurrence in MTS is inconsistent in the pediatric and adult literature. 12 14 35 Goldman et al reported a single patient in their cohort of patients with MTS, with loss of secondary stent patency due to congenital thrombophilia and poor adherence to anticoagulation. 14 Goldenberg et al reported 2 recurrent thromboses out of 6 patients with MTS to have associated hypercoagulability. 12 In contrast to the pediatric literature, a large retrospective analysis by Neglén et al in 870 adult patients did not show an increased risk for stent thrombosis in association with thrombophilia. 35 In our series, of the 5 patients with congenital thrombophilic risk factor, only 2 experienced recurrence/progression of thrombosis ( Table 1 ) at a median follow-up of 16.8 months (range 4.9–41.4 months); however, the generalizability of these findings is limited to the lack of uniform testing for hypercoagulability disorders in these patients (only 6 patients were tested).

Regardless for treatment modalities, these patients are at risk of developing PTS that is usually associated with swelling, pain, signs of venous insufficiency with dilated blood vessels, varicosities, skin changes, and potential for stasis ulceration in the long term, especially with recurrence of DVT or chronic venous insufficiency. 36 In one study, among adult patients with iliofemoral DVT who were managed with anticoagulation alone, 71% had leg swelling and 18% skin ulcerations at 10 years of follow-up. 37 In our study, signs and symptoms of PTS were clinically documented in 8 of the 13 patients who sustained a DVT; however, the grading severity could not be analyzed due to nonstandardized screening practices and documentation. A previously validated PTS survey tool was sent to all patients who experienced a DVT in the setting of PTS; however, this was met with a poor survey response rate (30.7%). Among patients with stent placement, 63% had clinically documented and/or self-reported PTS. With the exception of Murphy et al reporting 0% PTS symptoms in their cohort at last follow-up, our results are comparable to PTS rates reported by Goldman et al at 60% and Goldenberg et al at 75% of evaluable patients at last follow-up.

Although our study reports the largest pediatric cohort with MTS and real-life experience, we acknowledge several limitations that include retrospective study design, small sample size, heterogeneous treatment strategies, nonstandardized management and documentation, and recall bias. The clinical decision for CDT versus systemic thrombolysis, angioplasty, or stent placement, was at the discretion of the treating physician that incorporated patients' preferences as well. Due to nonstandardized screening methods during clinical documentation of PTS and poor response rate of the mailed-out survey, this study may underestimate the prevalence and severity of PTS.

In summary, MTS should be considered in adolescents with left iliofemoral thrombosis. CDT should be considered early on following diagnosis. For those patients who underwent anticoagulation with or without thrombolysis, no bleeding or procedural complications were observed. The decision algorithm for placement of endovascular stents in adolescents that have achieved full growth is not well established. Our institutional practice for stent placement takes into account the degree of vascular occlusion, resolution with anticoagulation and/or thrombolysis or thrombectomy, and vascular evidence of chronicity (e.g., presence of surrounding collateral vessels), to maximize the chances of successful endovascular stenting and prevent recurrent thrombosis or mechanical failure. Our study emphasizes the need of prospective multi-institutional collaborative efforts to study MTS in children and adolescents to establish standardized guidelines for diagnosis, management including anticoagulation, endovascular approach, and monitoring DVT-related sequelae to optimize long-term outcomes.
